# Thyroid and breast carcinomas in a patient with Pendred syndrome: a case report and literature review

**DOI:** 10.3389/fonc.2026.1593186

**Published:** 2026-01-30

**Authors:** Hongji Wu, Jie Xu, Ruzhong Xu, Linlin Shi, Lin Xu, Jin Zhou, Haitao Zheng

**Affiliations:** 1 Department of Thyroid Surgery, The Affiliated Yantai Yuhuangding Hospital of Qingdao University, Yantai, China; 2 Department of Clinical Nutrition, The Affiliated Yantai Yuhuangding Hospital of Qingdao University, Yantai, China; 3 Department of Pathology, The First Affiliated Hospital of Nanyang Medical College, Nanyang, China; 4 Department of Endocrinology and Foot & Ankle Surgery, The Affiliated Yantai Yuhuangding Hospital of Qingdao University, Yantai, China; 5 Key Laboratory Shandong Provincial Medicine and Health Key Laboratory of Endocrine and Metabolic Disease and Clinical Translational Medicine, Yantai, Shandong, China

**Keywords:** breast cancer, case report, Pendred syndrome, SLC26A4 gene, thyroid cancer

## Abstract

**Background:**

Goiter in the course of Pendred syndrome may in rare cases be associated with thyroid cancer (about 1% of all Pendred syndrome patients). The coexistence of Pendred syndrome with both thyroid and breast cancer is an even rarer condition reported in only one case.

**Case presentation:**

We report a case of a patient diagnosed with Pendred syndrome, who concurrently developed follicular thyroid cancer and breast cancer. After receiving four cycles of neoadjuvant THP (trastuzumab + pertuzumab + docetaxel) chemotherapy, the patient underwent left mastectomy and bilateral thyroidectomy. Postoperative pathology confirmed follicular thyroid carcinoma, while no residual malignancy was detected in the breast tissue. Genetic analysis of the SLC26A4 gene revealed mutations in intron 7 (c.919.2A>G) and exon 3 (c.170C>A) on chromosome 7. To minimize the risk of axillary metastasis, the patient received postoperative breast radiotherapy. At over one year of follow-up, there were no signs of recurrence for either cancer.

**Conclusions:**

The coexistence of Pendred syndrome with both thyroid and breast carcinoma is extremely rare, and the underlying mechanisms remain uncertain. Current evidence does not support *SLC26A4* as a driver oncogene, and the concurrence of malignancies may represent a coincidental finding rather than a causal association. Nonetheless, comprehensive genetic testing should be considered for patients with Pendred syndrome, and family-based screening is recommended once pathogenic *SLC26A4* variants are identified. Long-term surveillance of the thyroid and breast is essential for early detection and timely management of potential malignancies in these patients.

## Introduction

Pendred syndrome is an autosomal recessive disorder that is classically defined by the combination of sensorineural deafness/hearing impairment, goiter, and an abnormal organification of iodide with or without hypothyroidism (1). Vaughan Pendred was the first to report on the association of deafness and mut-ism and goiter occurring in two members of an Irish family in 1896 (2). Without reference to the Pendred report, Brain details five families and twelve children from the eastside of London in 1927 with co-occurrence of deafness, mutism, and goiter (3). All the affected persons had both goiter and deafness but were well developed physically and of normal intelligence. None of the parents was affected, which was suggestive for an autosomal recessive disorder. In 1958, Morgans and Trotter studied the iodine organification with the so-called perchlorate test and demonstrated that it was decreased in the goitrous thyroid tissue of affected individuals (4). A century after the initial description of the syndrome, two research groups identified the phenotype as localized to a region on chromosome 7q22-31.1 (5, 6). Shortly thereafter, in 1997, Everett identified the *SLC26A4* gene (originally named the PDS gene) responsible for Pendred syndrome by positional cloning and predicted that this gene encodes the protein pendrin (7). Pendrin functions as a chloride/bicarbonate exchanger in the inner ear and kidney, and is thought to mediate iodide efflux across the apical membrane of thyrocytes, a critical step for iodide organification and subsequent thyroid hormone synthesis (1). In total, about 600 mutations have been identified in the SCL26A4 gene to date. Over 100 of these are associated with Pendred syndrome (1, 8).

In Pendred syndrome, the thyroid-related disease primarily manifests as goiter, with some patients also experiencing hypothyroidism. The thyroid enlargement in Pendred syndrome develops typically during childhood and is variable both between and within families (9). It varies from slight thyroid enlargement to a large, diffuse or multinodular goiter (10). A single nodule may also be observed. However, according to references, goiter is not an essential symptom of Pendred syndrome and can be absent in almost 50% of cases (1, 8). Goiter in the course of Pendred syndrome may in rare cases be associated with thyroid cancer (about 1% of all Pendred syndrome patients) (11). Previously, there has only been one reported case of a patient with Pendred syndrome concurrently suffering from both thyroid cancer and breast cancer (12). This case does not provide a detailed explanation of the mutated genes, nor does it elucidate the potential relationship between these gene mutations and thyroid cancer or breast cancer. Here, a rare complication of thyroid and breast carcinomas with Pendred syndrome is described. We describe the mutation site of *SCL26A4* in this patient and explore the potential relationship between this gene mutation and thyroid cancer and breast cancer.

## Materials and methods

### Clinical data collection

Clinical information, imaging, surgical procedures, pathology, and follow-up data of the proband were obtained from the Department of Thyroid Surgery, Yantai Yuhuangding Hospital, Affiliated to Qingdao University. This case report was conducted in accordance with the Declaration of Helsinki. The Institutional Ethics Committee of Yantai Yuhuangding Hospital reviewed the case and determined that formal approval was not required because it involved anonymized clinical and genetic information. Written informed consent for genetic testing and publication was obtained from the patient and participating family members.

### Histological examination

Surgical specimens from the thyroid and breast were fixed in 10% neutral buffered formalin, embedded in paraffin, and sectioned at 4 μm. Sections were stained with hematoxylin and eosin (H&E) according to standard protocols. Slides were examined by two experienced pathologists to confirm the histological diagnosis.

### Immunohistochemistry

Tissue specimens were fixed in 10% neutral-buffered formalin at room temperature for 12 h, paraffin-embedded, and sectioned at 4 µm. Sections were deparaffinized and rehydrated at 60°C, then rinsed in PBS for 5 min. Antigen retrieval was performed in citrate buffer (pH 6.0) using microwave heating (5 min at high power, then 10 min at low power). Endogenous peroxidase was quenched with 3% hydrogen peroxide at 37°C for 10 min, followed by three PBS washes (5 min each). Primary antibodies (detailed information provided in **Supplementary Table 1**) were applied and incubated at 37°C for 60 min, followed by three PBS washes. A secondary antibody (Dako/Agilent Technologies, Santa Clara, CA, USA) was then applied at 37°C for 30 min, followed by three PBS washes. Color development was achieved with freshly prepared DAB for 8 min at 37°C. Slides were counterstained with hematoxylin for 10 min, dehydrated through graded ethanols, cleared in xylene, and coverslipped.

Interpretation adhered to contemporary consensus criteria. ER and PR were called positive when ≥1% of invasive tumor nuclei stained (per ASCO/CAP hormone receptor guidance) (13). HER2 was scored 0–3+ by IHC; 3+ was considered positive (uniform intense complete membrane staining in >10% of cells), whereas 2+ (weak–moderate complete membrane staining in >10% of cells) was considered equivocal and reflexed to ISH per the ASCO/CAP focused update algorithm; 0/1+ were negative (14). AR was interpreted using an institutional threshold aligned to ER/PR criteria (≥1% nuclear staining). For Ki-67, the labeling index was reported as the percentage of positive tumor nuclei after counting ≥1,000 cells in hotspot areas when feasible (≥500 as an absolute minimum in scant specimens), consistent with the International Ki67 Working Group’s analytic standardization recommendations (15). Other marker interpretation followed predefined laboratory SOPs and contemporary diagnostic practice (16). CK5/6, P63, CD56, Galectin-3, Synaptophysin, and CEA were considered positive when characteristic membranous and/or cytoplasmic staining was present in tumor cells per laboratory SOPs (generally using a ≥10% tumor-cell threshold); Pax-8, TTF-1, and Cyclin D1 required distinct nuclear staining. BRAF V600E IHC was recorded as positive/negative according to clone-specific manufacturer instructions and internal quality controls. All slides were independently reviewed by two board-certified pathologists, with discrepancies resolved by joint consensus.

### Whole-exome sequencing

Peripheral blood samples were subjected to whole-exome sequencing (WES). Genomic DNA was extracted using the DNeasy Blood & Tissue Kit (QIAGEN, Düsseldorf, North Rhine-Westphalia, Germany). Exonic regions were enriched using the TruSeq Exome Enrichment Kit (Illumina, San Diego, CA, USA), and libraries were prepared with the KAPA Hyper DNA Library Prep Kit (KAPA Biosystems, Wilmington, MA, USA) according to the manufacturer’s instructions. The targeted probe set (KingMed Diagnostics, Guangzhou, Guangdong, China) covered the exons, selected introns, and variable splice regions of 249 deafness-related genes (**Supplementary Table 2**). Sequencing was performed on the Illumina HiSeq 4000 platform (Illumina, San Diego, CA, USA). The mean sequencing depth of targeted exons and flanking 5 bp intronic regions exceeded 200×, with 99.8% of the target bases achieving ≥20× coverage. The sequencing experiment was established and validated by KingMed Diagnostics. Base calling was conducted using bcl2fastq (v2.16.0.10, Illumina Inc.) to generate FASTQ-formatted sequence reads (Illumina 1.8+ encoding). High-quality reads were aligned to the human reference genome (UCSC hg19, GRCh37) using the BWA-MEM algorithm (v0.7.12) with default parameters to produce SAM/BAM files. Secondary bioinformatic analysis was performed primarily using the Genome Analysis Toolkit (GATK, v3.4-0). Variant calling included single nucleotide variants (SNVs) and small insertions/deletions (indels). Classification of pathogenicity followed the American College of Medical Genetics and Genomics (ACMG) guidelines, integrating evidence from population databases (gnomAD), variant repositories (ClinVar, HGMD, dbSNP), and published literature (17, 18). Sequence variant nomenclature followed the Human Genome Variation Society (HGVS) guidelines. Variants were filtered based on the following criteria: (1) rare or novel variants; and (2) nonsense, frameshift, splice-site, or missense mutations. The integrated results from all bioinformatic tools were visually verified using the Integrative Genomics Viewer (IGV).

### Sanger sequencing validation

Candidate *SLC26A4* variants identified by WES were confirmed by Sanger sequencing. Genomic DNA from all available family members was extracted from 3 mL of peripheral blood using the QIAGEN Universal DNA Purification Kit. The primer pairs used for mutation verification were as follows: *SLC26A4* (c.919–2A>G), forward 5′-TGGAGTTTTTAACATCTTTTGTTTTATTCC-3′ and reverse 5′-TGGAGTTTTTAACATCTTTTGTTTTATTCC-3′; SLC26A4 (c.170C>A), forward 5′-TGGAGTTTTTAACATCTTTTGTTTTATTCC-3′ and reverse 5′-TGGAGTTTTTAACATCTTTTGTTTTATTCC-3′. PCR amplification was performed with an initial denaturation at 96°C for 1 min, followed by 25 cycles of 96°C for 10 s, 50°C for 5 s, and 60°C for 4 min. The PCR products were sequenced using an ABI 3730 Genetic Analyzer (Applied Biosystems, Foster City, CA, USA), and results were analyzed against the GRCh37/hg19 reference sequence.

### *In silico* prediction analyses

To evaluate the functional impact of the identified variants, multiple prediction tools were applied. For the splice-site variant c.919-2A>G, wild-type and mutant sequences encompassing exon 7 and approximately 100 bp of flanking intronic regions were submitted to NNSplice (19), NetGene2 (20, 21), and Human Splicing Finder (HSF) (22) to assess potential disruption of canonical splice acceptor or donor sites. For the nonsense variant c.170C>A (p.Ser57*), predictions were performed using MutationTaster2 (23), Combined Annotation Dependent Depletion (CADD v1.7) (24), and the Ensembl Variant Effect Predictor (VEP) (25) to estimate the likelihood of premature termination codon introduction, nonsense-mediated mRNA decay, and production of truncated nonfunctional protein.

## Patient findings

A 48-year-old woman presented with a palpable lump in her left breast that had persisted for two weeks. She had a medical history of congenital sensorineural deafness and thyroid enlargement but no prior history of cancer. On physical examination, a firm, mobile mass approximately 3 cm in diameter was noted in the upper inner quadrant of the left breast. There was no tenderness or nipple discharge. A palpable, firm, mobile lymph node measuring 3 cm was detected in the left axilla, with no tenderness. Examination of the neck revealed a grade III diffuse thyroid enlargement, but no palpable cervical lymph nodes.

**Figure 1:**
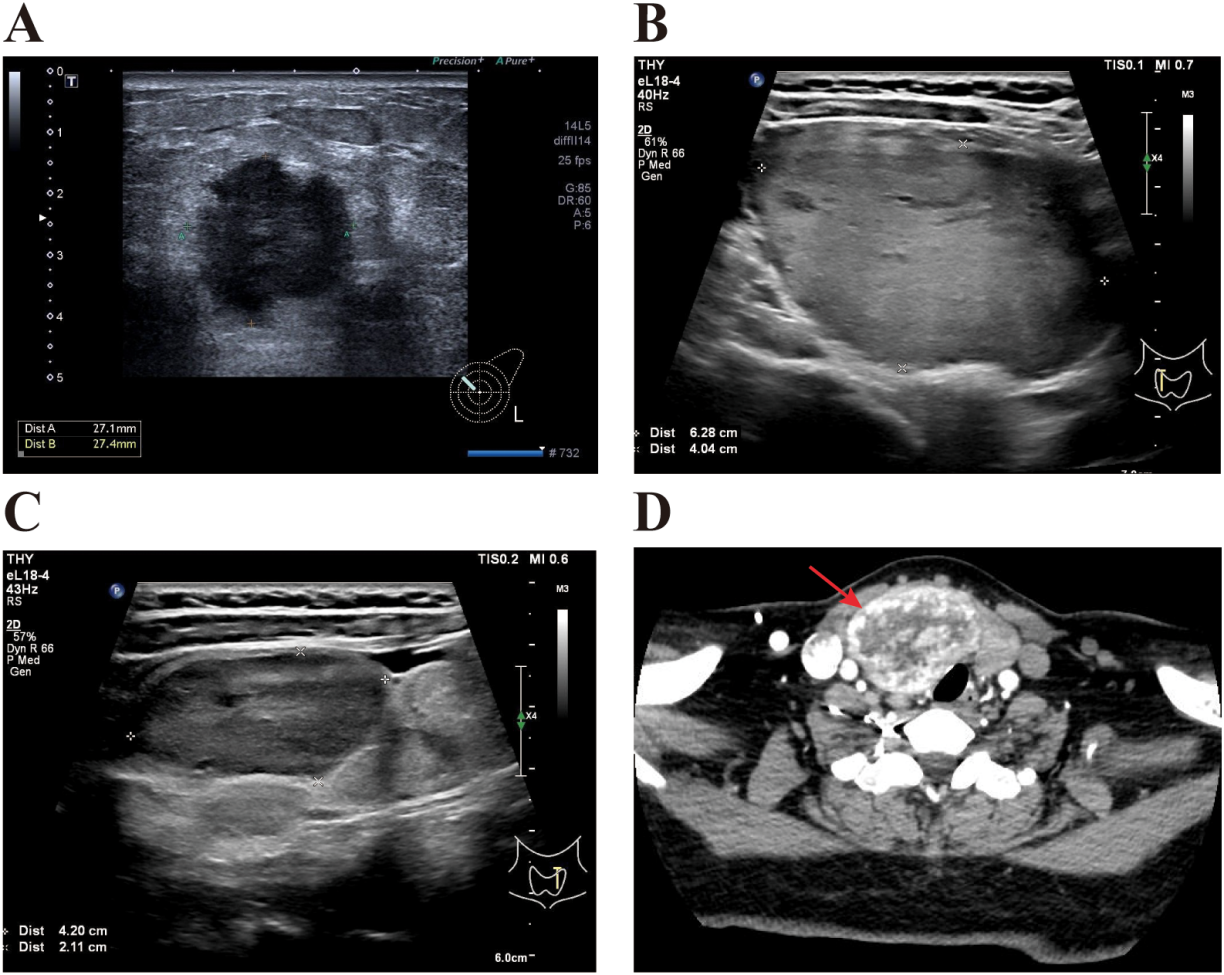
Preoperative ultrasound and computed tomography (CT) images of the breast and thyroid. **(A)** Doppler ultrasound of the breast and axilla shows a mass at the 10 o’clock position of the left breast, measuring 2.7×2.7 cm. The mass is highly suspicious for ductal carcinoma *in situ*, BI-RADS category 4c. **(B, C)** Doppler ultrasound of the thyroid and neck reveals multiple nodules in both thyroid lobes, with the largest on the right side measuring approximately 6.3×4.0 cm and on the left side measuring approximately 4.2×2.1 cm, both classified as TI-RADS 4a. **(D)** Thyroid CT scan shows a large mass on the right thyroid lobe compressing the trachea.

**Figure 2:**
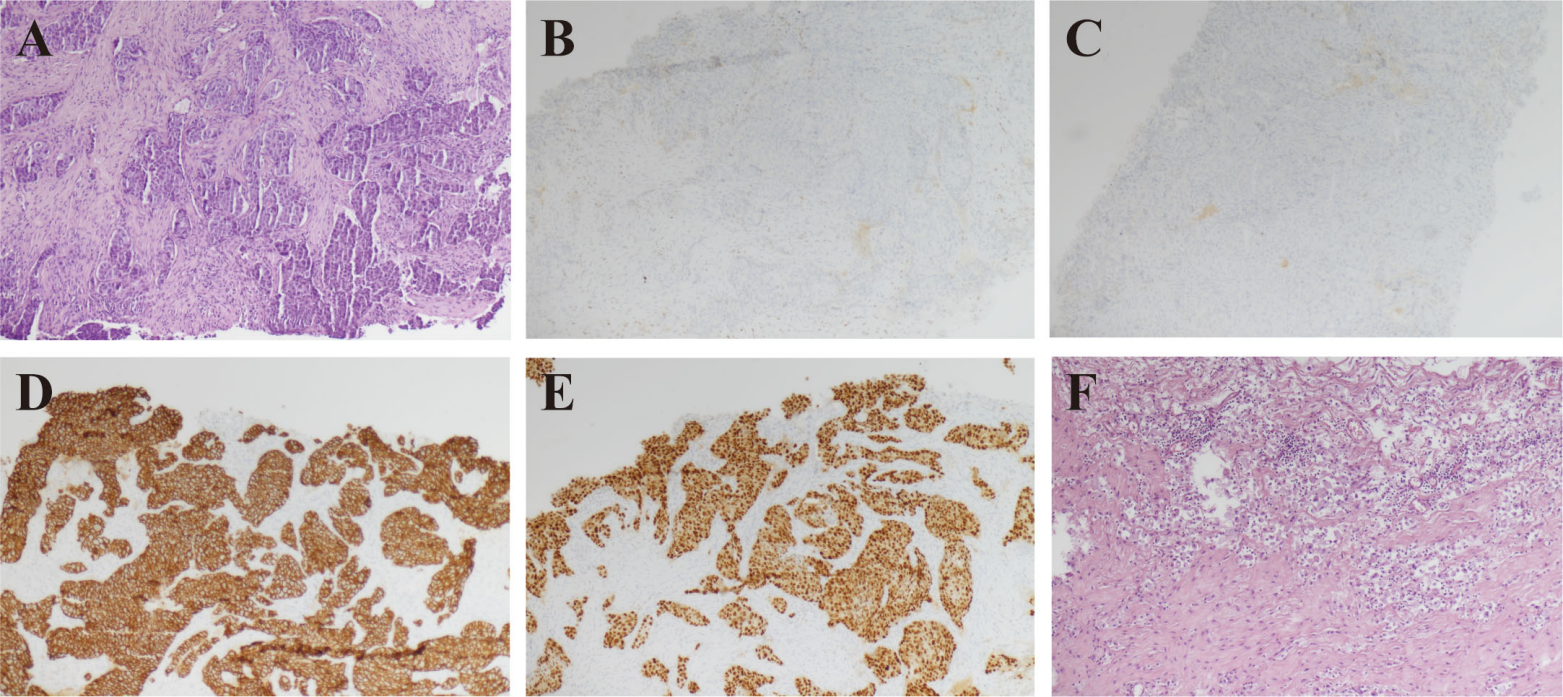
Preoperative and postoperative histological and immunohistochemical images of the left breast. **(A)** Microscopic examination with hematoxylin and eosin staining (×10) shows that the preoperative biopsy of the left breast mass is invasive carcinoma. **(B–E)** Immunohistochemical staining (×10) shows ER negative (-), PR negative (-), HER2 positive (3+), and AR positive (90%). **(F)** Hematoxylin and eosin staining (×10) of postoperative tissue analysis shows no malignant tumor, with only vascular elastic fiber stroma remaining.

The Doppler ultrasound test of the breast and axilla revealed a 2.7 × 2.7 cm lump located at the 10 o’clock position in the left breast, highly suspicious for intraductal carcinoma (BI-RADS 4c) (**Figure 1A**). The left axilla showed an enlarged lymph node measuring 2.6 × 1.8 cm. Fine-needle aspiration biopsy (FNAB) of the breast mass confirmed invasive ductal carcinoma, grade III. Immunohistochemistry results were estrogen receptor negative (−), progesterone receptor negative (−), HER2 positive (3+), androgen receptor 90% nuclei, CK5/6 negative (−), P63 negative (−), Bcl-2 positive (+), and Ki-67 labeling index 80% (**Figures 2A–E**). FNAB of the axillary lymph node showed no evidence of metastasis.

Given the size of the tumor and the imaging findings of multiple enlarged axillary lymph nodes, neoadjuvant therapy was initiated according to The NCCN Clinical Practice Guidelines in Oncology (NCCN Guidelines) for Breast Cancer (Version 3.2024) (26) and Chinese Society of Clinical Oncology Breast Cancer guidelines (CSCO Guidelines) 2024 (27). The patient received four cycles of trastuzumab, pertuzumab, and docetaxel (THP regimen). After neoadjuvant therapy, she opted for a left mastectomy and axillary lymph node dissection rather than oncoplastic or reconstructive surgery due to personal preference. The pathological examination of the resected breast tissue revealed a 1.7 × 1.5 × 1.5 cm gray-white fibrous area in the upper inner quadrant with no evidence of residual malignancy (**Figure 2F**). The examination of 14 excised axillary lymph nodes found no metastasis.

The patient also manifested a goiter which was not regularly monitored. The Doppler ultrasound examination of the thyroid and neck was performed because of progressive dyspnea suggestive of tracheal compression, revealing multiple TI-RADS 4a nodules. The largest nodules measured 6.3 × 4.0 cm in the right lobe and 4.2 × 2.1 cm in the left lobe (**Figures 1B, C**). The CT scan of the thyroid showed bilateral thyroid nodules with calcifications, with the larger right-sided nodule compressing the trachea(**Figure 1D**). Owing to its suspicious ultrasonographic features and compressive effect, FNAB of the dominant right thyroid nodule was performed and showed no evidence of malignancy. FNAB of the left lobe was discussed; however, as the patient already had a clear surgical indication and a total thyroidectomy with intraoperative frozen-section examination was planned, the result would not have altered the surgical strategy, and the patient declined an additional FNAB after informed counseling. Serum thyroglobulin (TG) was markedly elevated (>500 ng/mL; reference range: 3.5–77 ng/mL). Other thyroid function tests, including thyroid-stimulating hormone (TSH), free thyroxine (FT4), free triiodothyronine (FT3), thyroid peroxidase antibody (TPOAB), and thyroglobulin antibody (TGAB), were within normal limits(**Supplementary Table 3**). However, urinary iodine concentration was markedly increased (1787.1 μg/L; reference range: 100–300 μg/L), indicating iodine enrichment.

**Figure 3:**
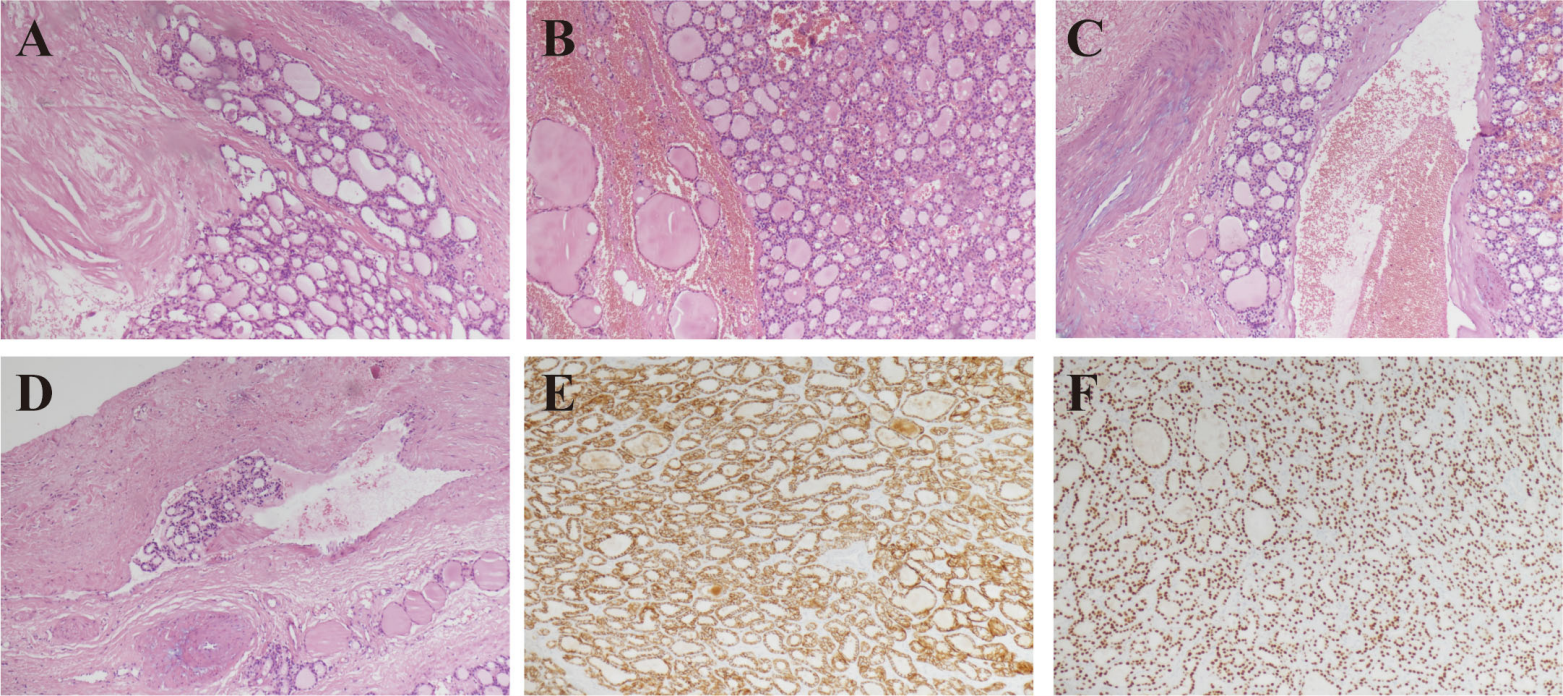
Histological and immunohistochemical images of bilateral thyroid follicular carcinoma postoperatively. Microscopic examination with hematoxylin and eosin staining (×10) shows left thyroid follicular tumor with capsular invasion **(A)** and extracapsular nodule **(B)**. Right thyroid follicular tumor shows extracapsular nodule, vascular invasion **(C)**, and tumor thrombus in the vessel **(D)**. **(E, F)** Immunohistochemical staining (×10) shows CD56 positive (+) and TTF-1 positive (+).

Given the suspicious imaging findings, the patient underwent total thyroidectomy with intraoperative frozen section evaluation. Frozen section pathology revealed follicular neoplasms in both lobes. Final paraffin pathology confirmed follicular thyroid carcinoma in both lobes, with capsular invasion and extracapsular nodules on the left and vascular invasion with intravascular tumor thrombi on the right. Immunohistochemistry results were CK19 negative (−), Synaptophysin negative (−), GATA-3 negative (−), CEA negative (−), Galectin-3 negative (−), Pax-8 positive (+), CD56 positive (+), TTF-1 positive (+), Cyclin D1 positive (+), and BRAF V600E negative (−). (**Figure 3**)

**Figure 4:**
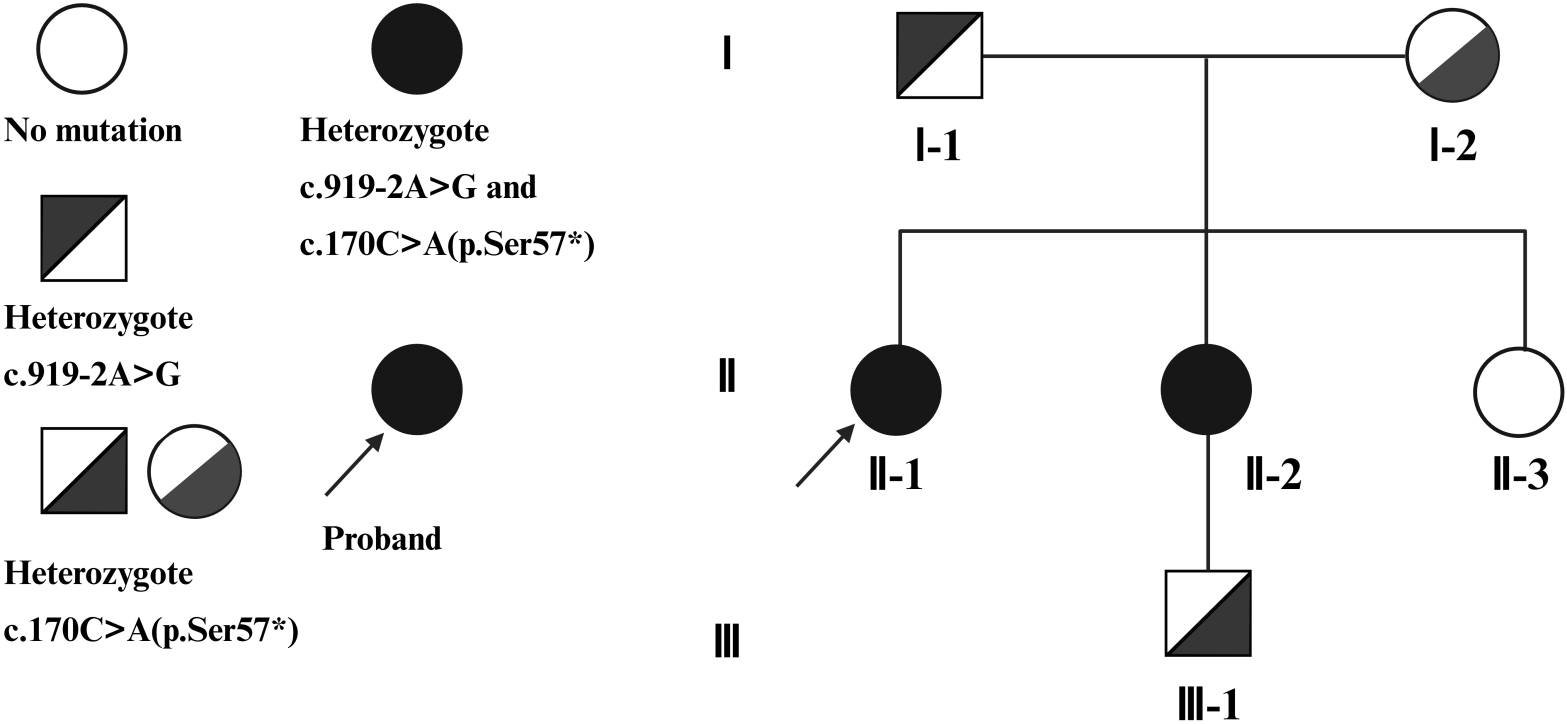
Family pedigree showing the inheritance of *SLC26A4* mutations. The proband (II-1) and her sister (II-2) both carry compound heterozygous mutations, c.919-2A>G (intron 7) and c.170C>A (p.Ser57*) (exon 3). The father (I-1) carries the heterozygous c.919-2A>G mutation, and the mother (I-2) carries the heterozygous c.170C>A (p.Ser57*) mutation. The proband’s other sister (II-3) died in early childhood, and her genetic status is unknown. The proband’s sister’s son (III-1) carries the heterozygous c.170C>A (p.Ser57*) mutation. Created in BioRender. Wu, H. (2025) https://BioRender.com/3zhski8.

Considering the concurrent thyroid enlargement and congenital deafness, Pendred syndrome was suspected. Inner ear imaging of the proband revealed inner ear malformation with an enlarged vestibular aqueduct (EVA). Peripheral venous blood samples were collected, and genomic DNA was analyzed using next-generation sequencing (NGS), followed by confirmation by Sanger sequencing. Genetic analysis identified compound heterozygous mutations in the *SLC26A4* gene in the proband (**Figure 4**, II-1): c.919-2A>G (intron 7) and c.170C>A (p.Ser57*) (exon 3).

Subsequently, genomic DNA from all available family members was analyzed using NGS and confirmed by Sanger sequencing. In addition, all available relatives underwent preliminary audiometric screening and thyroid physical examination, followed by thyroid function testing, including TSH, FT4, FT3, Tg, TPOAb, and TgAb. All results were within the reference ranges, indicating preserved thyroid function in these family members (**Supplementary Table 4**). The proband’s younger sister (**Figure 4**, II-2) carried the same compound heterozygous mutations and presented with congenital hearing loss and goiter, similar to the proband. Unlike the proband, she had no thyroid or breast nodules, which distinguished her clinical phenotype from that of the proband. Their father (**Figure 4**, I-1) carried a heterozygous c.919-2A>G mutation, while their mother (**Figure 4**, I-2) carried a heterozygous c.170C>A (p.Ser57*) mutation; neither parent showed thyroid abnormalities or hearing loss. Another sister (**Figure 4**, II-3) died in early childhood of an unknown cause, and her genetic status is unknown. The proband’s sister’s son (**Figure 4**, III-1) carried the heterozygous c.170C>A (p.Ser57*) mutation but was clinically unaffected. We recommended further thyroid ultrasonography and inner ear MRI for comprehensive evaluation in all relatives. However, the proband’s parents (I-1, I-2) and nephew (III-1) declined additional imaging, as they exhibited no Pendred syndrome–related symptoms. The proband’s younger sister (II-2) also declined further examinations due to financial constraints, despite our strong recommendation for thyroid ultrasound and inner ear MRI.

The distribution of mutations within the family is illustrated in the pedigree (**Figure 4**), and representative Sanger sequencing chromatograms are provided in **Supplementary Figure S1**. In silico prediction analysis indicated that the c.919-2A>G variant abolishes the canonical 3′ splice acceptor site (**Table 1**), while the c.170C>A (p.Ser57*) variant is predicted to result in premature termination and loss of function(**Table 2**).

**Table 1:** In silico splicing predictions for the *SLC26A4* variant c.919-2A>G.

Predictor tool	Sequence/Transcript (reference: GRCh37/hg19)	WT prediction	MUT prediction	Interpretation
NNSplice	200 bp genomic sequence surrounding c.919-2A>G (ENST00000644269.2_4, ± 100 bp)	Canonical acceptor detected, score ≈ 0.93	No acceptor site detected	Loss of canonical acceptor site
NetGene2	Same 200 bp genomic sequence (ENST00000644269.2_4)	Acceptor site predicted, confidence ≈ 0.72	No acceptor site detected	Loss of canonical acceptor site
HSF (Human Splicing Finder Pro)	ENST00000644269 [NM_000441], HGVS input c.919-2A>G	Acceptor site at position 107683443, score 90.03, motif GTTTTATTTCAGAC	Acceptor site at position 107683443, score 62.15, motif GTTTTATTTCGGAC (*Broken WT Acceptor Site*)	Significant reduction of splice site strength; canonical acceptor abolished

WT, wild type; MUT, mutant. Scores are prediction confidence values provided by each algorithm. All three tools consistently predicted that c.919-2A>G abolishes the canonical 3′ splice acceptor site of *SLC26A4*, strongly suggesting aberrant splicing.

**Table 2:** In silico predictions for the *SLC26A4* nonsense variant c.170C>A (p.Ser57*).

Predictor tool	Sequence/Transcript (reference: GRCh37/hg19)	Prediction/Score	Interpretation
MutationTaster2	Gene symbol: *SLC26A4*; Transcript: ENST00000265715 (NM_000441, 4930 bases, protein coding); Variant input: c.170C>A (SNV position: 170, base change: C>A)	Predicted deleterious; automatic classification due to nonsense-mediated decay (NMD)	Variant introduces a premature stop codon (p.Ser57*), expected to cause loss of function via NMD
CADD v1.7	Chromosome: 7; Position: 107303746; Ref: C; Alt: A; Model: GRCh37-v1.7	Raw score = 9.90; PHRED-scaled score = 41	Highly deleterious (scores >20 suggest pathogenicity)
Ensembl VEP	Assembly: GRCh37.p13; Input: NM_000441.2:c.170C>A	Consequence: *stop_gained (p.Ser57*); annotated as pathogenic in ClinVar/HGMD	Nonsense mutation leading to truncated protein; classified as pathogenic

WT, wild type; MUT, mutant; NMD, nonsense-mediatedmRNA decay. MutationTaster2, CADD, and Ensembl VEP consistently predict that c.170C>A (p.Ser57*) is a deleterious nonsense variant, highly likely to abolish protein function.

Postoperatively, the patient underwent adjuvant radiation therapy to reduce the risk of axillary recurrence. She was also prescribed lifelong levothyroxine for thyroid hormone replacement. In accordance with current ATA recommendations for differentiated thyroid cancer with at least intermediate risk and no evidence of persistent disease, levothyroxine therapy was titrated with the aim of maintaining serum TSH in a moderately suppressed range of approximately 0.1–0.5 mIU/L rather than allowing full normalization (28). At a follow-up of two years post-breast surgery and 19 months post-thyroid surgery, no recurrence or metastasis was observed. Ultrasound imaging of the breast, thyroid, and associated lymph nodes showed no abnormalities. During postoperative follow-up, serum TSH values initially increased (16.5–16.9 mIU/L) and later decreased to 0.41–0.56 mIU/L. At the most recent visit in February 2024, TSH was 4.49 mIU/L. The patient experienced no postoperative complications such as lymphedema, hoarseness, or hypocalcemia.

## Discussion

There has been only one reported case of a Pendred syndrome patient concurrently suffering from both thyroid cancer and breast cancer, which published in 1998 (12). However, that report lacked genetic testing and did not explore the mechanisms underlying tumor development. Given the rarity of such cases, the potential mechanisms remain poorly understood. Nevertheless, available evidence allows exploration of the underlying correlations and pathogenesis.

Clinical manifestations of Pendred syndrome classically include congenital sensorineural deafness, goiter, and abnormal iodide metabolism with or without hypothyroidism (8). Goiter typically develops at puberty, often manifesting as multinodular, although Sreekar et al. showed that the thyroid can retain a normal size in some Pendred syndrome patients, while in others, giant goiters with retrosternal extension (29). The prevalence of Pendred syndrome has been estimated at 7.5–10 per 100,000 (9). With advances in genetic testing more cases are being diagnosed, yet cases involving progression to thyroid malignancy are extremely rare. To date, 12 cases of thyroid cancer coexisting with Pendred syndrome have been reported (10). Pendred syndrome is associated with an increased risk of non-medullary thyroid tumors, particularly follicular thyroid cancer and the follicular variant of papillary thyroid cancer (10). Rarely, it may progress to anaplastic thyroid cancer (30).

Goiter is a common thyroid manifestation in patients with Pendred syndrome. In the thyroid, pendrin is expressed on the apical membrane of follicular cells and mediates the efflux of iodide from the thyrocyte into the follicular lumen, where iodide is organified and incorporated into thyroid hormones (31). Mutations in SLC26A4 impair pendrin function, resulting in defective iodide efflux and organification, inefficient thyroid hormone synthesis, and compensatory TSH stimulation that gradually drives follicular cell hypertrophy and hyperplasia, ultimately contributing to diffuse or multinodular goiter (32, 33). Animal experiments have demonstrated that excessive intake of iodine downregulates pendrin expression and promotes goiter (32). In Pendred syndrome, impaired iodide efflux reduces the efficiency of thyroid hormone synthesis. Over time, the pituitary may maintain TSH at relatively higher levels within the physiological range or with intermittent elevation, resulting in chronic stimulation of thyroid follicular cells. Although such “relative” TSH stimulation is not universal and was not clearly present in our patient, it has been proposed as one of several mechanisms—together with iodine excess—that may promote nodular growth and facilitate malignant transformation of follicular cells (30). Although many patients with Pendred syndrome remain clinically euthyroid, the reported prevalence of subclinical or overt hypothyroidism varies widely across studies. Earlier cohort studies estimated a frequency of approximately 30–50%, predominantly subclinical hypothyroidism; however, rates as high as 79% have been described in populations with insufficient iodine intake (34–36). In contrast, studies from iodine-replete countries such as Japan and Korea have reported predominantly euthyroid phenotypes (34). These observations indicate that thyroid function in Pendred syndrome is strongly influenced by environmental iodine exposure and may differ substantially across populations. Hypermethylation of the *SLC26A4* gene is also reported to be an early event in the development of thyroid tumors. Meanwhile, expression of pendrin in thyroid cancers is lower than in normal thyroid tissue (37). Thus, studying Pendred cases with nodular thyroid lesions may clarify how pendrin, TSH, and iodine dysregulation contribute to malignant transformation.

An important issue in this case was how to interpret the markedly elevated serum thyroglobulin level. Although follicular carcinoma can produce thyroglobulin (Tg) because it arises from Tg-secreting follicular cells, it is important to emphasize that elevated serum Tg is not specific for thyroid cancer. In clinical practice, Tg measurement is primarily used for postoperative surveillance of differentiated thyroid cancer after total thyroidectomy to monitor residual or recurrent disease, and its routine use for initial diagnosis is not recommended because it lacks sufficient specificity (38). In the present case, the markedly elevated preoperative Tg level likely reflected a combination of factors, including the large thyroid tissue burden associated with a significantly enlarged, multinodular gland and excessive iodine exposure, with additional contribution from the follicular carcinoma itself. Tg reflects the amount of thyroid tissue present regardless of underlying pathology, and elevated levels can be observed in various benign conditions, such as benign thyroid nodules, autoimmune thyroid disease, and iodine excess (39). The substantial decrease in Tg to very low values after total thyroidectomy suggests effective removal of Tg-producing thyroid tissue, including both normal and malignant components. However, this postoperative decrease does not allow definitive inference regarding the sole preoperative source of Tg elevation, as both benign thyroid tissue and tumor tissue were resected. Taken together, these findings underscore that preoperative Tg elevation may be multifactorial and is not sufficiently specific to serve as a standalone diagnostic marker for malignancy. Although preoperative Tg measurement is not routinely recommended as a diagnostic test because of its limited specificity, we decided to measure Tg before surgery in this patient for clinical reasons. Preoperative Tg may provide useful contextual information regarding the overall thyroid tissue burden and potential tumor load at presentation, and, more importantly, it establishes a baseline value that allows meaningful interpretation of serial Tg measurements after surgery (40). Patients with normal or low Tg before surgery may not show a significant postoperative rise in Tg even in the presence of recurrent disease, whereas those with markedly elevated Tg preoperatively are more likely to demonstrate rising Tg if recurrence develops. Therefore, obtaining a preoperative Tg value may help avoid false reassurance during postoperative surveillance and facilitates individualized long-term follow-up.

Another important aspect in this case is how to interpret the markedly elevated urinary iodine concentration. Urinary iodine concentration (UIC) is widely used as a surrogate marker of recent iodine intake. Recent systematic reviews indicate that iodine deficiency moderately increases the risk of developing thyroid nodules, whereas more than adequate or excessive iodine intake does not show a consistent effect on nodule risk (41). In contrast, several case–control studies and a meta-analysis have reported that excessive iodine intake, defined as UIC ≥300 μg/L, is positively associated with the occurrence of papillary thyroid carcinoma (PTC), while adequate iodine intake (100–199 μg/L) may be protective (42). However, the available data are heterogeneous, and a causal relationship between iodine excess and thyroid carcinogenesis has not been firmly established. In our patient, the UIC of 1,787.1 μg/L clearly reflects extreme iodine excess and may have contributed to thyroid tissue stress in the setting of Pendred syndrome. Nevertheless, based on current evidence, we consider iodine excess more likely as a co-factor that may have facilitated an already vulnerable thyroid milieu, rather than as a direct and sufficient cause of carcinogenesis.

A further key consideration in this case was how to manage the appropriate degree of TSH suppression after total thyroidectomy. According to current ATA recommendations, patients with differentiated thyroid carcinoma who fall within at least an intermediate-risk category and have no evidence of persistent disease are generally managed with moderately suppressed TSH levels (approximately 0.1–0.5 mIU/L), rather than allowing complete normalization (28). In our patient, postoperative TSH values were initially markedly elevated, most likely reflecting suboptimal levothyroxine replacement in the early follow-up period. With subsequent titration, TSH declined toward the recommended range; however, a mild elevation later reappeared, highlighting the need for continued monitoring and individualized dose adjustment over time. Taken together, these observations emphasize that TSH suppression strategies should balance oncologic risk reduction with avoidance of overtreatment and should be reassessed dynamically during long-term follow-up.

The relationship between Pendred syndrome and breast cancer has not been clearly reported. Nevertheless, a biologically plausible connection has been proposed through disruption of iodide transport in the mammary gland. Pendrin is endogenously expressed in mammary tissue, mediating Na^+^-independent iodide transport. Mutations in *SLC26A4* may impair pendrin folding, localization, or transport, thereby limiting the availability of iodide to breast tissue. Because iodide has been reported to exert antioxidant and antiproliferative effects, reduced iodide delivery may theoretically weaken these protective mechanisms and contribute to tumor development. Anguiano et al. also demonstrated that a moderately high concentration of iodine intake can alleviate the symptoms of mammary fibrosis in women, can reduce the occurrence of chemically induced mammary cancer in rats (50–70%), and can mediate anti-proliferation and apoptosis events in MCF-7 cells (43). Furthermore, in cell experiments, pendrin, and not sodium-iodide symporter (NIS), was found to play a vital role in the transport of iodine in the mammary gland (44). When expression of pendrin is compromised, its iodine transport function is decreased, thereby increasing the risk of mammary cancer. In a recent study, low levels of protein and mRNA of pendrin were detected in breast cancer (45). Taken together, these results provide a possible explanation for the development of mammary cancer in patients with Pendred syndrome. Recent research has proposed that iodine (particularly in its iodide form) may exert protective effects by acting as an antioxidant and by mediating antineoplastic, antiproliferative, and pro-apoptotic activities in several cancer models (46). Consequently, disruption of iodide transport in Pendred syndrome could theoretically diminish these protective effects and thereby contribute to tumorigenesis.

Pendred syndrome is an autosomal recessive disorder, with biallelic pathogenic mutations required for clinical manifestations. Genetic analysis demonstrated that the proband harbored compound heterozygous variants in *SLC26A4*: a canonical splice-acceptor variant, c.919-2A>G (intron 7), inherited from the father, and a nonsense variant, c.170C>A (p.Ser57*) (exon 3), inherited from the mother. The c.919-2A>G variant alters the invariant −2 nucleotide of the splice acceptor site and is predicted to disrupt normal splicing, potentially resulting in exon skipping or activation of cryptic splice sites, leading to abnormal or absent pendrin protein. This variant has been reported in multiple cohorts of hearing-impaired patients and segregates with disease in several families (47, 48), and is observed only in heterozygous state in East Asian individuals in gnomAD (≈101 carriers, no homozygotes). The c.170C>A variant introduces a premature stop codon at residue 57 and is expected to cause loss of function via nonsense-mediated decay or truncated protein. It has been detected in affected individuals (49, 50) and is rare in population databases (gnomAD heterozygotes n = 3, no homozygotes).

It has been reported that the most common mutation in SLC26A4 in the Chinese population is C.919-2A>C, followed byC.2168A>G. Patients carrying both of these allelic mutations have exhibited severe phenotypes (51). Importantly, the nonsense mutation c.170C>A (p.Ser57*) identified in the present family has previously been reported only in patients from Pakistan, Iran, and the Uygur population (49, 52, 53), and more recently in Turkish patients with hereditary hearing loss (54). Moreover, Danilchenko et al. described three Tuvan patients from Southern Siberia carrying the same compound heterozygous variants (c.919-2A>G and c.170C>A), highlighting that this genotype combination is not unique to our family and providing additional support for its pathogenic role (55). To our knowledge, however, the present study represents the first report of this variant combination in Han Chinese individuals, expanding the mutational and ethnic spectrum associated with SLC26A4-related disorders.

In silico prediction supported the pathogenicity of both variants. For c.919-2A>G, NNSplice identified a strong acceptor site in the wild-type sequence that was completely abolished in the mutant; NetGene2 also predicted loss of the canonical acceptor; and Human Splicing Finder indicated a significant reduction in acceptor strength (from 90.03 to 62.15). For c.170C>A (p.Ser57*), MutationTaster2 classified the variant as deleterious through introduction of a premature termination codon, CADD yielded a high PHRED-scaled score of 41, and Ensembl VEP annotated it as a pathogenic stop-gain variant in ClinVar/HGMD. To provide an integrated overview, **Table 3** summarizes the two identified *SLC26A4* variants, their database annotations, population allele frequencies, and predicted effects derived from *in silico* analyses. According to ACMG/AMP guidelines, both variants meet the criteria for pathogenic classification, supported by PVS1 (canonical splice/nonsense variant), PM2 (extremely low frequency in gnomAD), PP1 (segregation in affected family members), and PP4 (phenotype highly specific for Pendred syndrome).

**Table 3:** Summary of *SLC26A4* variants identified in the family.

Variant	Location	Molecular consequence	dbSNP	ClinVar	gnomAD AF	Effect
c.919-2A>G	Intron 7	Splice acceptor variant	rs111033313	Pathogenic	0.00036	Loss of canonical splice site
c.170C>A (p.Ser57*)	Exon 3	Nonsense variant	rs111033200	Pathogenic	0.00001	Premature stop codon → NMD/truncated protein

ClinVar and dbSNP identifiers were obtained from the NCBI databases. Allele frequencies are derived from the Genome Aggregation Database (gnomAD v2.1.1). “Effect” summarizes the predicted functional consequences based on in silico analyses presented in **Tables 1** and **2**.

Because Pendred syndrome is recessive, clinical manifestations were present only in the proband and her sister who carried compound heterozygous variants. In contrast, carriers of a single heterozygous variant (father, mother, and nephew) remained unaffected, consistent with autosomal recessive inheritance. This underscores the value of comprehensive family-based genetic testing for identifying carriers and providing genetic counseling. Although comprehensive family screening is recommended in Pendred syndrome, implementation in real-world practice is often challenging. In our case, several asymptomatic relatives declined further thyroid ultrasonography and inner ear MRI, either because they perceived no relevant symptoms or due to financial constraints. This highlights an important but frequently overlooked issue: even when genetic risk is clearly identified, acceptance of recommended surveillance may remain limited, potentially delaying early detection of thyroid or cochlear abnormalities.

Functionally, both variants are expected to cause marked reduction or loss of pendrin expression in thyroid follicular cells and mammary tissue, impairing iodide efflux, promoting chronic TSH stimulation, goiter formation, and potentially altering redox biology. Such mechanisms provide plausible biological links to thyroid tumorigenesis and possibly breast pathology. However, the direct role of *SLC26A4* loss-of-function in carcinogenesis remains speculative. Further functional studies—including RT-PCR of patient RNA to confirm splicing disruption by c.919-2A>G, quantitative assays of pendrin expression, and iodide-transport experiments with truncated protein—are needed to clarify the molecular consequences and tumorigenic potential of these variants.

Although previous studies have reported that *SLC26A4* mutations may impair the folding, localization, or transmembrane transport of pendrin, thereby limiting iodide uptake and potentially linking such alterations to the development of thyroid or breast cancer, no evidence to date has clearly identified pendrin as a driver oncogene in either malignancy. Therefore, in the present case, we consider the coexistence of *SLC26A4* mutations and malignancy to be more likely a rare coincidence rather than a direct causal relationship. It is possible that the patient harbored additional pathogenic variants or driver mutations responsible for the development of thyroid and breast cancer, or other oncogenic alterations shared by both tumor types—such as mutations in PIK3CA—that were not detected in this study (56). This hypothesis may also explain why the proband’s younger sister, who carried the same *SLC26A4* mutations, did not develop thyroid or breast cancer, suggesting that the proband may possess additional undetected pathogenic variants contributing to her distinctive clinical phenotype.

Furthermore, the proband’s sister exhibited classical features of Pendred syndrome—congenital hearing loss and goiter—consistent with the presence of the same compound heterozygous *SLC26A4* mutations. However, unlike the proband, she did not develop any malignant lesions, which may be attributed to the absence of additional oncogenic alterations predisposing to thyroid or breast cancer. This observation further supports the notion that the coexistence of malignancies in the proband is unlikely to be directly caused by *SLC26A4* mutations. Instead, these mutations alone may be insufficient to drive tumorigenesis, and other genetic or molecular events may underlie the patient’s concurrent thyroid and breast cancers.

This study has several limitations that should be acknowledged. First, the findings are based on a single family case, which limits the generalizability of our conclusions. Functional validation experiments—such as *in vitro* assays to assess pendrin expression, localization, and iodide transport capacity—were not performed, and thus the pathogenic impact of the identified *SLC26A4* variants remains to be experimentally confirmed. Moreover, the targeted sequencing panel used in this study focused on deafness-related genes and did not comprehensively cover all potential oncogenic drivers. Therefore, it is possible that additional pathogenic or driver mutations contributing to thyroid and breast tumorigenesis were not detected. Although previous reports have suggested a potential link between *SLC26A4* dysfunction and tumor development, current evidence does not support pendrin as a bona fide driver oncogene. The coexistence of *SLC26A4* mutations and malignancy in this patient may therefore represent a rare coincidence rather than a causal relationship. Future studies integrating whole-genome or transcriptome sequencing and functional analyses are warranted to elucidate the molecular mechanisms underlying such phenotypic coexistence.

## Conclusion

We report a rare case of Pendred syndrome accompanied by both thyroid carcinoma and breast carcinoma, in which compound heterozygous *SLC26A4* mutations (c.919–2A>G and c.170C>A) were identified. These findings highlight the uncommon coexistence of Pendred syndrome caused by *SLC26A4* mutations with two distinct malignancies. However, current evidence does not support *SLC26A4* as a relevant driver oncogene, and the concurrence of these malignancies may represent a rare coincidence rather than a causal relationship. Further comprehensive genomic and functional studies are warranted to elucidate the underlying mechanisms of concurrent thyroid and breast carcinomas.

## Data Availability

The original contributions presented in the study are included in the article/**Supplementary Material**. Further inquiries can be directed to the corresponding authors.

## Supplementary material

Supplementary Table 1 Detailed information on the primary antibodies used for immunohistochemistry.

Supplementary Table 2 List of 249 deafness-related genes included in the targeted capture panel used for whole-exome sequencing (WES).

Supplementary Table 3 Thyroid function parameters during follow-up with reference ranges. TSH, thyroid-stimulating hormone; FT4, free thyroxine; FT3, free triiodothyronine; TPOAb, thyroid peroxidase antibody; TgAb, thyroglobulin antibody; Tg, thyroglobulin. “–” indicates parameters that were not measured at the corresponding follow-up visit.

Supplementary Table 4 Clinical and laboratory findings of family members. N, normal; TSH, thyroid-stimulating hormone; FT3, free triiodothyronine; FT4, free thyroxine; TPOAb, thyroid peroxidase antibody; TgAb, thyroglobulin antibody; Tg, thyroglobulin; ↑, elevated.

Supplementary Figure 1 Sanger sequencing chromatograms of *SLC26A4* gene mutations in the proband and family members. The proband (II-1) and her sister (II-2) both show Het mutations for c.919-2A>G and c.170C>A (p.Ser57*), confirmed by both forward and reverse sequencing. The father (I-1) has a Het c.919-2A>G mutation, with forward sequencing results. The mother (I-2) has a Het c.170C>A (p.Ser57*) mutation, with reverse sequencing results. The proband’s sister (II-3) is deceased, and genetic testing was not performed. The proband’s sister’s son (III-1) carries the Het c.170C>A (p.Ser57*) mutation, confirmed by both forward and reverse sequencing. Het, Heterozygous; WT, Wild type.
