# Supplementary material for: Thyroid and breast carcinomas in a patient with Pendred syndrome: a case report and literature review
Source: Front Oncol. 2026 Jan 30;16:1593186. doi: 10.3389/fonc.2026.1593186 (PMC12900729; doi:10.3389/fonc.2026.1593186)
Supplement: Supplementary Table 1 — Detailed information on the primary antibodies used for immunohistochemistry. [file Table1.docx]

**SUPPLEMENTARY TABLE 1. Detailed information on the primary antibodies used for immunohistochemistry**

| **Primary antibody** | **Company** | **Catalog Number** | **Clone Number** | **IHC dilution ratio** |
| --- | --- | --- | --- | --- |
| **ER** | Proteintech | 84564-4-RR | 241825D5 | 1:1000 |
| **PR** | Proteintech | 85112-4-RR | 242686G1 | 1:1000 |
| **HER2** | Proteintech | 60311-1-Ig | 1B12A7 | 1:1600 |
| **AR** | Proteintech | 66747-1-Ig | 1F7C12 | 1:8500 |
| **CK5/6** | Proteintech | 68295-1-Ig | 1F9E1 | 1:12000 |
| **p63** | Proteintech | 60332-2-Ig | 4D6G12 | 1:5000 |
| **Bcl-2** | Proteintech | 60178-1-Ig | 4H8C6 | 1:15000 |
| **Ki-67** | Proteintech | 84192-4-RR | 241499E7 | 1:2500 |
| **CK19** | Proteintech | 60187-1-Ig | 3G1E4 | 1:26000 |
| **Synaptophysin** | Proteintech | 67864-1-Ig | 4E12C4 | 1:1000 |
| **GATA-3** | Proteintech | 66400-1-Ig | 1B11A8 | 1:250 |
| **CEA** | Proteintech | 68377-1-Ig | 2B8H6 | 1:5000 |
| **Galectin-3** | Proteintech | 60207-1-Ig | 1C1B2 | 1:2000 |
| **Pax-8** | Proteintech | 60145-4-Ig | 4H7B3 | 1:10000 |
| **CD56** | Proteintech | 60238-1-Ig | 1E8C9 | 1:5000 |
| **TTF-1** | Proteintech | 66034-1-Ig | 2C8F3 | 1:250 |
| **Cyclin D1** | Proteintech | 82681-1-RR | 2G3G5 | 1:1000 |
| **BRAF V600E** | Abcam | ab228461 | VE1 | 1:100 |
