# Supplementary material for: Thyroid and breast carcinomas in a patient with Pendred syndrome: a case report and literature review
Source: Front Oncol. 2026 Jan 30;16:1593186. doi: 10.3389/fonc.2026.1593186 (PMC12900729; doi:10.3389/fonc.2026.1593186)
Supplement: Supplementary Table 2 — List of 249 deafness-related genes included in the targeted capture panel used for whole-exome sequencing (WES). [file Table2.docx]

**SUPPLEMENTARY TABLE 2. List of 249 deafness-related genes included in the targeted capture panel used for whole-exome sequencing (WES)**

| ABHD12 | ABHD5 | ACTB | ACTG1 | ADCY1 | ADGRV1 | AIFM1 |
| --- | --- | --- | --- | --- | --- | --- |
| ALMS1 | AMMECR1 | ANKH | ARSG | ASAH1 | ATP1A3 | ATP2B2 |
| ATP6V1B1 | ATP6V1B2 | BCAP31 | BCS1L | BDP1 | BSND | BTD |
| CABP2 | CACNA1D | CATSPER2 | CCDC50 | CD151 | CD164 | CDC14A |
| CDH23 | CDK9 | CDKN1C | CEACAM16 | CEP250 | CEP78 | CHD7 |
| CHSY1 | CIB2 | CISD2 | CLDN14 | CLIC5 | CLPP | CLRN1 |
| COCH | COL11A1 | COL11A2 | COL1A1 | COL1A2 | COL2A1 | COL4A3 |
| COL4A4 | COL4A5 | COL4A6 | COL9A1 | COL9A2 | COL9A3 | CRYL1 |
| CRYM | DCAF17 | DCDC2 | DIABLO | DIAPH1 | DIAPH3 | DLX5 |
| DMXL2 | DNMT1 | DSPP | EDN3 | EDNRB | EIF3F | ELMOD3 |
| EPS8 | EPS8L2 | ERCC2 | ERCC3 | ESPN | ESRP1 | ESRRB |
| EXOSC2 | EYA1 | EYA4 | FAM136A | FAM189A2 | FDXR | FGF3 |
| FGFR1 | FGFR2 | FGFR3 | FITM2 | FLNA | FOXC1 | FOXI1 |
| GAA | GALNS | GATA3 | GIPC3 | GJA1 | GJB1 | GJB2 |
| GJB3 | GJB6 | GPRASP2 | GPSM2 | GRHL2 | GRXCR1 | GRXCR2 |
| GSDME | HARS1 | HARS2 | HGF | HMX1 | HOMER2 | HOXA2 |
| HOXB1 | HSD17B4 | IFNLR1 | ILDR1 | KARS1 | KCNE1 | KCNJ10 |
| KCNQ1 | KCNQ4 | KIT | KITLG | LARS2 | LHFPL5 | LHX3 |
| LMX1A | LOXHD1 | LRP2 | LRTOMT | MAN2B1 | MANBA | MARVELD2 |
| MASP1 | MCM2 | MET | MGP | MIR96 | MITF | MPZ |
| MPZL2 | MSRB3 | MYH14 | MYH9 | MYO15A | MYO1A | MYO3A |
| MYO6 | MYO7A | NARS2 | NDP | NDRG1 | NEFL | NF2 |
| NLRP3 | OPA1 | OSBPL2 | OTOA | OTOF | OTOG | OTOGL |
| P2RX2 | PAX3 | PCDH15 | PDE1C | PDZD7 | PEX1 | PEX26 |
| PEX6 | PISD | PJVK | PMP22 | PNPT1 | POLR1C | POLR1D |
| POU3F4 | POU4F3 | PROK2 | PROKR2 | PRPS1 | PTPN11 | RDX |
| RIPOR2 | RMND1 | ROR1 | RPGR | RPS6KA3 | S1PR2 | SALL1 |
| SALL4 | SEMA3E | SERPINB6 | SIX1 | SIX5 | SLC17A8 | SLC19A2 |
| SLC22A4 | SLC26A4 | SLC26A5 | SLC29A3 | SLC33A1 | SLC4A11 | SLC52A2 |
| SLC52A3 | SLITRK6 | SMAD4 | SMPX | SNAI2 | SOX10 | SOX2 |
| SPATA5 | STAG2 | STRC | SUCLA2 | SUCLG1 | SYNE4 | SYT2 |
| TBC1D24 | TBL1X | TBX1 | TCIRG1 | TCOF1 | TECTA | TFAP2A |
| TIMM8A | TJP2 | TMC1 | TMEM126A | TMEM132E | TMIE | TMPRSS3 |
| TMPRSS5 | TNC | TPRN | TRIOBP | TRMU | TSHZ1 | TSPEAR |
| TUBB4B | TWNK | TYR | USH1C | USH1G | USH2A | VCAN |
| WBP2 | WFS1 | WHRN | XYLT2 |  |  |  |
