# Supplementary material for: Thyroid and breast carcinomas in a patient with Pendred syndrome: a case report and literature review
Source: Front Oncol. 2026 Jan 30;16:1593186. doi: 10.3389/fonc.2026.1593186 (PMC12900729; doi:10.3389/fonc.2026.1593186)
Supplement: Supplementary Table 3 — Thyroid function parameters during follow-up with reference ranges. TSH, thyroid-stimulating hormone; FT4, free thyroxine; FT3, free triiodothyronine; TPOAb, thyroid peroxidase antibody; TgAb, thyroglobulin antibody; Tg, thyroglobulin. “–” indicates parameters that were not measured at the corresponding follow-up visit. [file Table3.docx]

**SUPPLEMENTARY TABLE 3. Thyroid function parameters during follow-up with reference ranges.**

| **Date** | **TSH (mIU/L)** | **FT4 (pmol/L)** | **FT3 (pmol/L)** | **TPOAb (IU/mL)** | **TgAb (IU/mL)** | **Tg (ng/mL)** |
| --- | --- | --- | --- | --- | --- | --- |
| **Reference range** | 0.27–4.20 | 12–22 | 3.1–6.8 | 0–34 | 0–115 | 3.5–77 |
| 2022-05-05  (preoperative) | 0.657 | 19.59 | 5.27 | 15.2 | 35 | >500 ↑ |
| 2022-06-12 | 16.51 ↑ | 14.96 | 2.85 | – | – | – |
| 2022-08-24 | 16.9 ↑ | 13.4 | 3.38 | – | 18.5 | 0.154 |
| 2022-11-15 | 0.413 | 22.3 ↑ | 5.31 | – | 11.8 | <0.04 |
| 2023-05-30 | 0.56 | 19.6 | 3.95 | 9.13 | 12.6 | – |
| 2024-02-01 | 4.49 ↑ | 17.6 | 4.18 | – | 13.2 | 0.111 |

Abbreviations: TSH, thyroid-stimulating hormone; FT4, free thyroxine; FT3, free triiodothyronine; TPOAb, thyroid peroxidase antibody; TgAb, thyroglobulin antibody; Tg, thyroglobulin. “–” indicates parameters that were not measured at the corresponding follow-up visit.
