# Supplementary material for: Thyroid and breast carcinomas in a patient with Pendred syndrome: a case report and literature review
Source: Front Oncol. 2026 Jan 30;16:1593186. doi: 10.3389/fonc.2026.1593186 (PMC12900729; doi:10.3389/fonc.2026.1593186)
Supplement: Supplementary Table 4 — Clinical and laboratory findings of family members. N, normal; TSH, thyroid-stimulating hormone; FT3, free triiodothyronine; FT4, free thyroxine; TPOAb, thyroid peroxidase antibody; TgAb, thyroglobulin antibody; Tg, thyroglobulin; ↑, elevated. [file Table4.docx]

**Supplement Table 4. Clinical and laboratory findings of family members**

| **Member** | **Relationship** | **Hearing assessment** | **Thyroid physical examination** | **TSH/FT3/FT4** | **TPOAb/TgAb** | **Tg** |
| --- | --- | --- | --- | --- | --- | --- |
| **II-1** | Proband | Deafness | Bilateral diffuse thyroid enlargement with palpable nodules in both lobes | N | N | ↑ |
| **II-2** | Sister | Deafness | Bilateral diffuse thyroid enlargement without distinct nodules | N | N | N |
| **I-1** | Father | N | N | N | N | N |
| **I-2** | Mother | N | N | N | N | N |
| **III-1** | Sister’s son | N | N | N | N | N |

Abbreviations: N, normal; TSH, thyroid-stimulating hormone; FT3, free triiodothyronine; FT4, free thyroxine; TPOAb, thyroid peroxidase antibody; TgAb, thyroglobulin antibody; Tg, thyroglobulin; ↑, elevated.
