# Supplementary figures and images for: Thyroid and breast carcinomas in a patient with Pendred syndrome: a case report and literature review
Source: Front Oncol. 2026 Jan 30;16:1593186. doi: 10.3389/fonc.2026.1593186 (PMC12900729; doi:10.3389/fonc.2026.1593186)

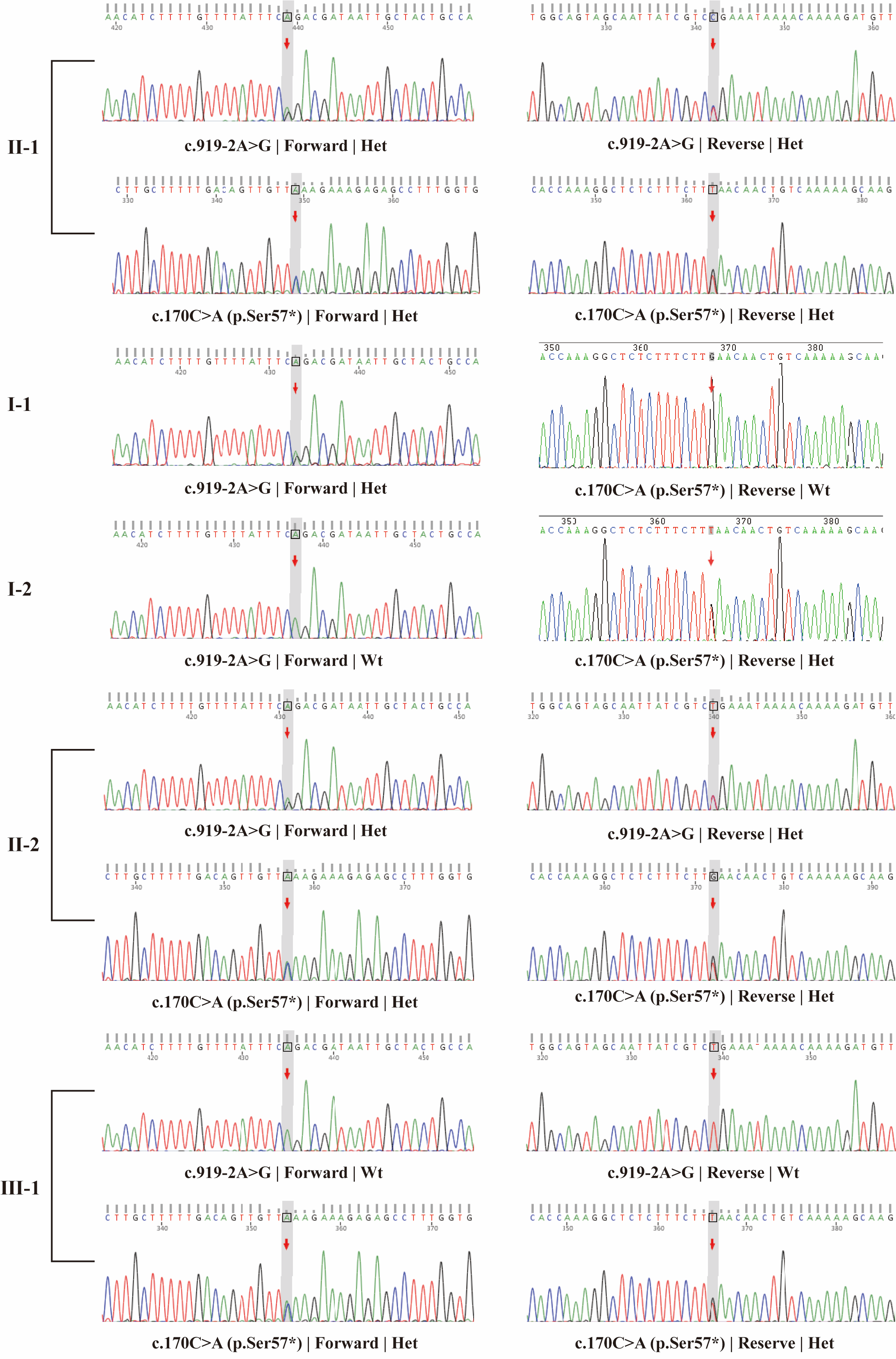

Supplement: Supplementary Figure 1 — Sanger sequencing chromatograms of SLC26A4 gene mutations in the proband and family members. The proband (II-1) and her sister (II-2) both show Het mutations for c.919-2A>G and c.170C>A (p.Ser57*), confirmed by both forward and reverse sequencing. The father (I-1) has a Het c.919-2A>G mutation, with forward sequencing results. The mother (I-2) has a Het c.170C>A (p.Ser57*) mutation, with reverse sequencing results. The proband’s sister (II-3) is deceased, and genetic testing was not performed. The proband’s sister’s son (III-1) carries the Het c.170C>A (p.Ser57*) mutation, confirmed by both forward and reverse sequencing. Het, Heterozygous; WT, Wild type. [file Image1.tif]
